# Laser Shock Peening of SiCp/2009Al Composites: Microstructural Evolution, Residual Stress and Fatigue Behavior

**DOI:** 10.3390/ma14051082

**Published:** 2021-02-26

**Authors:** Rujian Sun, Ziwen Cao, Yongxin Zhang, Hepeng Zhang, Yingwei Yu, Zhigang Che, Junfeng Wu, Shikun Zou, Wei Guo

**Affiliations:** 1 Science and Technology on Power Beam Processes Laboratory, AVIC Manufacturing Technology Institute, Beijing 100024, China; svip@163.com (Z.C.); meczgang@sina.com (Z.C.); wjf88813@163.com (J.W.); zoushk@sina.com (S.Z.); 2 Aviation Key Laboratory of Science and Technology on Advanced Surface Engineering, AVIC Manufacturing Technology Institute, Beijing 100024, China; 3 School of Mechanical Engineering and Automation, Beihang University, Beijing 100191, China; yongxinzhang@buaa.edu.cn (Y.Z.); hepeng_zhang86@163.com (H.Z.); gwei@buaa.edu.cn (W.G.); 4 AVIC Changhe Aircraft Industry (Group) Corporation LTD., Jingdezhen 333002, China; yuyingweihold@163.com

**Keywords:** discontinuously reinforced metal matrix composite, fatigue behavior, laser shock peening, microstructural evolution, residual stress

## Abstract

SiC particle reinforced aluminum alloy has a wide application in the aerospace industries. In this study, laser shock peening (LSP), an advanced surface modification technique, was employed for SiCp/2009Al composite to reveal its microstructure, microhardness and residual stress evolution. After peening, high densities of dislocations were induced in the aluminum substrate, and stacking faults were introduced into the SiC particle. The microhardness was increased from 155–170 HV to 170–185 HV, with an affected depth of more than 1.5 mm. Compressive residual stresses of more than 200 MPa were introduced. The three-point bending fatigue of the base material, laser peened and milled after laser peened specimens with artificial crack notch fabricated by a femtosecond laser was investigated. The average fatigue lives of laser peened and milled after laser peened specimens were increased by up to 10.60 and 2.66 times, compared with the base material. This combined fundamental and application-based research seeks to comprehensively explore the applicability of LSP on metal matrix composite.

## 1. Introduction

SiC particle reinforced aluminum is a typical discontinuously reinforced metal matrix composite, which has significant applications in manufacturing components and parts for aerostructures, such as ventral fins (SiC/6092Al/17.5p, SiC particle reinforced 6092 aluminium alloy with a particle volume fraction of 17.5%), panel supports (SiC/6061Al/25p) and helicopter blade sleeves (SiC/2009Al/15p) [1], due to their excellent balance of specific strength and stiffness, as well as the urgent demand for weight savings [2,3,4]. However, these metal matrix composite manufactured aerostructures are frequently subjected to severe mechanical loads, thermal stresses and cyclic vibration during operation [5], which are likely to cause a great reduction in their fatigue life and seriously affect their use reliability. Hence, worldwide attention has been paid to further improve the application-based properties of these metal matrix composites. The main methods include composition design (particle size, weight fraction and distribution) [6,7,8], particle addition [9,10,11], metallurgy process optimization [12], post-heat treatment [13,14] and coating deposition [15]. However, the above-mentioned methods are commonly complex, inefficient in mass production and time-consuming for engineering implementation.

Laser shock peening (LSP), an advanced surface treatment technique, has the unique capacity in introducing high-amplitude and large-depth compressive residual stress into the surface layer, thus improving the fatigue, wear and corrosion resistance for components [16,17,18]. Since the early invention of this technique in the 1970s [19], it has been primarily applied to various metals and alloys [20,21,22,23,24], and it has been implemented in the production environment for over two decades. Thus, it was not until the year 2006 that the LSP was explored to treat engineering ceramics [25,26]. After that, research was carried out on polycrystalline cubic boron nitride [27], ZrO_2_-based ceramic [28], Al_2_O_3_ ceramics [29] and SiC ceramics [30], which enable LSP techniques to be potentially used for ceramic surface modification. The successful LSP application in both metal and ceramic makes it a possible alternative for the surface modification of metal matrix composite. However, relevant research on this topic to date is insufficient. The parameter optimization for peening, the strengthening mechanism of LSP on metal matrix composite and its further property evaluation must be enriched.

In this study, LSP experiments were carried out on a SiC particle reinforced 2009 aluminum (SiCp/2009Al) composite. The microstructure evolution, microhardness and residual stress were carefully studied. Thus, the fatigue behavior of three-point bending was further investigated. This work seeks to gain a comprehensive understanding of the surface modification of metal matrix composite and aims to explore the applicability of LSP on composites.

## 2. Materials and Methods

### 2.1. Materials

**Figure 1:**
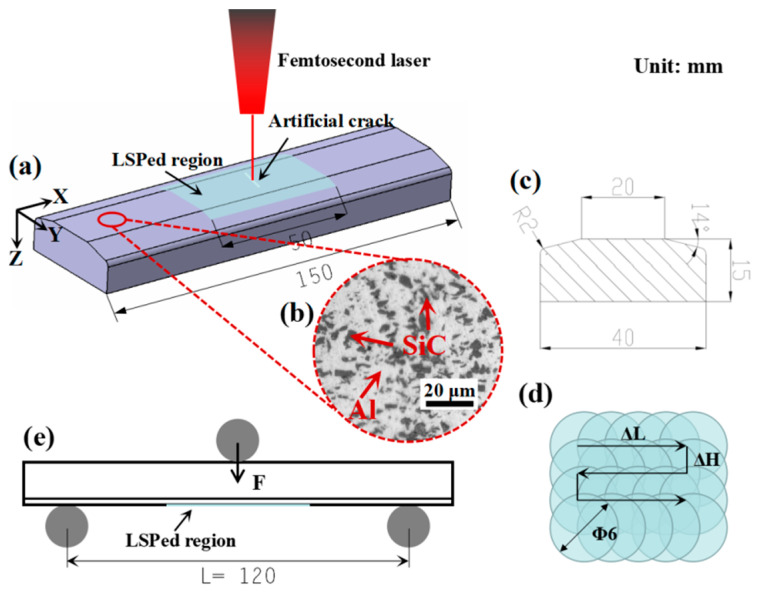
(**a**) Three-dimensional illustration of the preparation of 150 mm three-point bending fatigue specimens with the laser shock peened region indicated by light blue pattern and the artificial crack demonstrated by the white line; (**b**) optically observed microstructure of the SiCp/2009Al composite employed in this study, where the black irregulars are SiC particles and the rest are the 2009 aluminum substrate; (**c**) cross-sectional view of the three-point bending fatigue specimen including the detailed dimensions; (**d**) zigzag laser path illustrating an overlapping rate 40%; and (**e**) the experimental setup of the three-point bending fatigue test, where the support span is 120 mm.

The SiCp/2009Al composite was obtained from AVIC Changhe Aircraft Industry (Group) Corporation LTD. (Jingdezhen, China), which has a SiC volume fraction of 15%, as indicated by the black irregulars in Figure 1b. Thus, the chemical compositions of the 2009 aluminum substrate are given in Table 1. The yield strength, ultimate tensile strength and Young’s modulus of this SiCp/2009Al composite are 367 MPa, 550 MPa and 95 GPa, respectively.

### 2.2. LSP and Femtosecond Laser Experiments

LSP experiments were conducted at AVIC Manufacturing Technology Institute (Beijing, China) on a Q-switched Nd: YAG high power-pulsed laser system with a wavelength of 1064 nm, a pulse duration of 15 ns (full width at half maximum, FWHM), a circular spot size of 6 mm in diameter and an overlapping rate of 40% (corresponding to a ΔL = ΔH = 2.4 mm). The employed laser pulse energy was 20 and 30 J, corresponding to a power density of 4.72 and 7.08 GW/cm^2^, respectively. Prior to LSP, a 3M (Saint Paul, MN, USA) manufactured 100-μm-thick aluminum foil was attached to the peening surface to avoid possible damage or roughening of the irradiated surface and to reduce the reflection loss of the incident laser pulse. During LSP, a running deionized water layer with a thickness of 1–2 mm was applied as a transparent constrained layer to increase the peak pressure of the laser-induced shock wave. Specimens were peened twice with a zigzag laser path (see Figure 1d) covering an area of approximately 50 mm (X direction) × 25 mm (Y direction), as shown by the light blue pattern in Figure 1a. The length in the Y direction enables that the 14° edge can be fully covered. After LSP, a 10 mm artificial crack was initiated on the central line of each specimen using a high energy femtosecond laser system (PHAROS, Light conversion, Vilnius, Lithuania) with a wavelength of 1030 nm, a pulse duration of 200 fs, a power of 400 mW, a frequency of 10 kHz and a travel speed of 100 mm/min. The manufactured crack initiation had a size around 10 mm × 50 μm × 30 μm (length × width × depth).

### 2.3. Three-Point Bending Fatigue Tests

Specimens for the three-point bending fatigue test were designed according to the methodology adopted from T. E. Pistochini and M. R. Hill [31]. The fatigue specimens had a total length of 150 mm, and their detailed dimensions were illustrated in the cross-sectional view (see Figure 1c). Additionally, the surface of the fatigue specimens was milled to a roughness of Ra 1.6 μm. The specimens were divided into three types: base material (BM); twice laser shock peened (TLSPed); and milled after twice laser shock peened (MATLSPed). The 20 and 30 J twice laser shock peened were named TLSPed-20 J and TLSPed-30 J, while the specimens milled after 20 and 30 J twice laser shock peened were classified as MATLSPed-20 J and MATLSPed-30 J.

The three-point bending fatigue tests were performed on a high-frequency fatigue machine (QBG-100, Qianbang, Changchun, China), which can automatically find an appropriate frequency itself according to the geometry of the specimens. Tests for BM, LSPed and MALSPed were all carried out under ISO 7438: 2020 metallic materials—bend test) [32]. The specimens were positioned on the bottom supports with a span of 120 mm, and the external applied force was loaded at the centerline on the other side. The stress amplitude *σ_a_* can be approximately calculated according to the following equation:

(1) $$\sigma_{a}=\frac{3FL}{2bh^{2}}$$

where *F* is the applied force; *L* is the span of bottom supports, which is 120 mm; *b* is the specimen width, which is 40 mm; *h* is the sample height, which is 15 mm; and *σ_a_* is the maximum stress amplitude (MPa). Here, in our study, a sinusoidal external force of 16.8 kN (corresponding to a stress amplitude of 336 MPa) was applied for the test with a stress ratio of 0.1.

### 2.4. Material Characterizations

The microstructure evolution was observed using a scanning electron microscope (SEM, Gemini 500, Carl Zeiss, Oberkochen, Germany) and a transmission electron microscope (TEM, JEM-2100F, JEOL, Tokyo, Japan). The ultra-thin BM and TLSPed chips for TEM observation were both prepared by focused ion beam milling, as the conventional electrolytic jet thinning and argon ion beam polishing failed to prepare a suitable TEM observation region on the center of the chips. A scanning transmission electron microscope (STEM) coupled with energy dispersive X-ray spectroscopy (EDS) was also evolved to map the chemical compositions of specific phases. It is noteworthy that only the TLSPed-30 J specimen was employed for preparing the TLSPed TEM observation chips, as we deemed that even though there would be a difference in the TEM observation results between the TLSPed-20 J and TLSPed-30 J specimens, it would predictably lie in the density of the dislocations and the dislocation morphologies. Yet, this paper does not focus on revealing the different microstructural evolutions with the peening parameter but explores the microstructural evolution between the BM and the TLSPed ones.

The microhardness evolution in the depth direction was measured on the cross-section of the BM and TLSPed specimens with a test weight of 100 g and a dwell time of 10 s. It was measured from the top surface to a depth of 1.5 mm with a step distance of 0.05 mm. To eliminate the deviation, each measurement was conducted three times, and only average values were presented.

The residual stress was measured on the surface of the TLSPed and MATLSPed specimens based on the non-destructive X-ray diffraction technique on the LXRD system (PROTO, Ontario, Canada). It was measured using a Cr anode through {311} plane at a 2θ angle of 139°. These measurements were repeated three times on each specimen.

## 3. Results and Discussion

### 3.1. Microstructure Evolution

**Figure 2:**
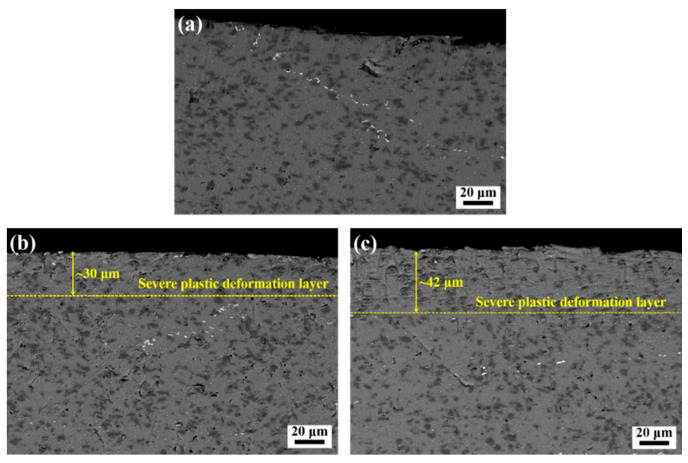
Scanning electron microscope (SEM) images illustrating the cross-sectional microstructures in the (**a**) base material (BM), (**b**) TLSPed-20 J and (**c**) TLSPed-30 J specimens.

Figure 2 shows the SEM images of the cross-sectional microstructures observed in the BM, TLSPed-20 J and TLSPed-30 J specimens. Compared with the microstructure in the BM specimen (see Figure 2a), severe plastic deformation was generated after peening, with the thickness of the severe plastic deformation layer being ~30 μm (see Figure 2b) and ~42 μm (see Figure 2c) in the TLSPed-20 J and TLSPed-30 J specimens, respectively. The generation of such plastic deformation was the result of the intensive interaction between the laser-induced shock wave and the surface material. Apart from the deformation layer, SEM cannot provide any further information about the microstructural evolutions.

**Figure 3:**
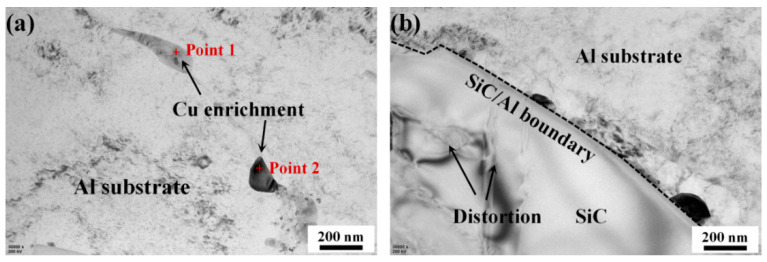
Typical transmission electron microscope (TEM) images illustrating microstructural morphology in the BM specimen. (**a**) The original aluminum substrate with Cu enrichment and (**b**) initial SiC particle with slight distortion inside and crack free SiC/Al boundary.

Figure 3 shows the TEM images of the original microstructure in the BM specimen. It can be seen from Figure 3a that neither an obvious dislocation nor mechanical twin structure was generated in the aluminum substrate, as the typical annealing was applied after the free forging [33,34]. Only slight Cu enrichment can be detected, where the chemical compositions indexed by EDS mapping are listed in Table 2. The possible phases for Point 1 and Point 2 were Al_3_Cu_2_ and AlCu solid solution, respectively. Figure 3b demonstrates the morphology of initial SiC particles. Slight distortion can be observed inside the particles, which can be attributed to the compression of the surrounding aluminum substrate during cooling. Additionally, a crack-free SiC/Al boundary can be seen as well.

**Figure 4:**
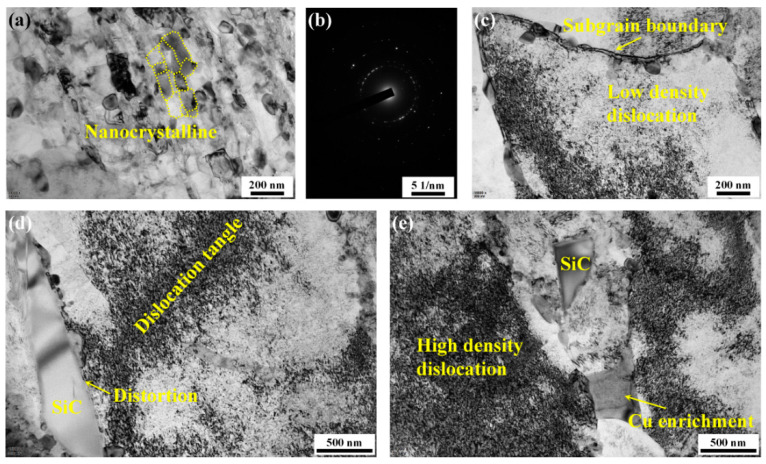
Typical TEM images illustrating microstructural morphology of aluminum substrate in the TLSPed-30 J specimen. (**a**) Nanocrystals generated on the peened surface, (**b**) corresponding SAED pattern of concentric rings, (**c**) low density of dislocation, (**d**) dislocation tangles and distortion at the SiC/Al boundary and (**e**) high density of dislocations.

Figure 4 depicts the microstructural evolution of the aluminum substrate in the TLSPed-30 J specimen. As can be seen from Figure 4a,b, nanocrystals were induced on the laser peened surface. This could have resulted from the intensive interaction between the laser-induced shock wave and the material surface. Additionally, multiple peening times also contributed. As described in [35], the peak pressure of the laser-induced shock wave is proportional to the square root of reduced shock impedance and laser power density, expressed as follows:

(2) $$P_{\max}(GPa)=0.01\sqrt{\frac{\alpha }{2\alpha +3}}\sqrt{Z(g\cdot cm^{-2}\cdot s^{-2})}\sqrt{I_{0}(GW\cdot cm^{-2})}$$

where *α* is the efficiency of the interaction, *Z* is the reduced shock impedance between the material and the confining medium and *I*_0_ is the laser power density. Hence, the peak pressure of the 30 J laser pulse-induced shock wave was 2.71 GPa, which is significantly higher than the dynamic yield strength of the SiCp/2009Al composite [36]. During LSP, the surface material underwent a rapid compressive deformation, and dislocations were induced. Their further tangling, movement, separating and rotating formed smaller new grains. When the grains were fined down to a nanometer scale, the nanocrystals were generated. Figure 4b is the corresponding pattern of the selected area electron diffraction (SAED) taken from Figure 4a, which is characterized by several concentric rings. The occurrence of such ring patterns can be seen as a typical feature for nanocrystals, which is widely reported in works of literature [37,38].

Figure 4c–e show the different dislocation structures induced in the aluminum substrate during the shock wave and material interaction. Dislocation tangles can be observed, while no obvious dislocation lines can be detected. This is because the surface layer was so severely deformed during the multiple LSP process that it was difficult to find a zone with clear dislocation line structures. Additionally, the varying density in dislocation zones is related to the attenuation of the laser-induced shock wave pressure, as the lower peak pressure can only drive a lower degree of atom layer distortion, resulting in the generation of a smaller number of dislocations. It is also worth noting that distortion was generated at the SiC/Al boundary, which is due to the discontinuity of the atom layer at the boundary.

**Figure 5:**
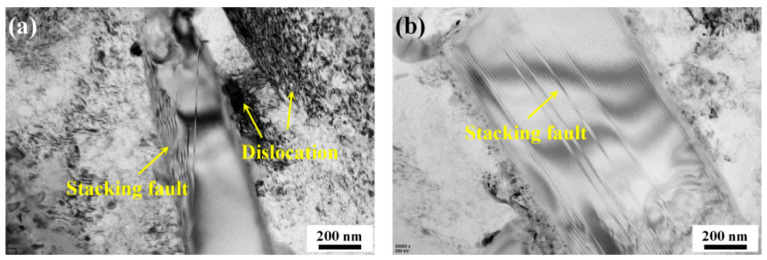
Typical TEM images illustrating microstructural morphology of SiC particles in the TLSPed-30 J specimen. (**a**) Stacking fault generated near the SiC/Al boundary, and (**b**) stacking fault generated inside the SiC particle.

Figure 5 illustrates the microstructure evolution of the SiC particles in the TLSPed-30 J specimen. Figure 5a,b demonstrate the occurrence of stacking faults. This is because the SiC particles employed are in an α-based hexagonal closest packed (hcp) structure, which only has three independent slip systems. According to the Von-Mises rule, five independent slip systems are necessary to maintain plastic deformation compatibility [39,40]. Hence, the deformation of SiC during LSP is difficult to generate, and only a short distance slide occurred. Therefore, the stacking fault was detected.

**Figure 6:**
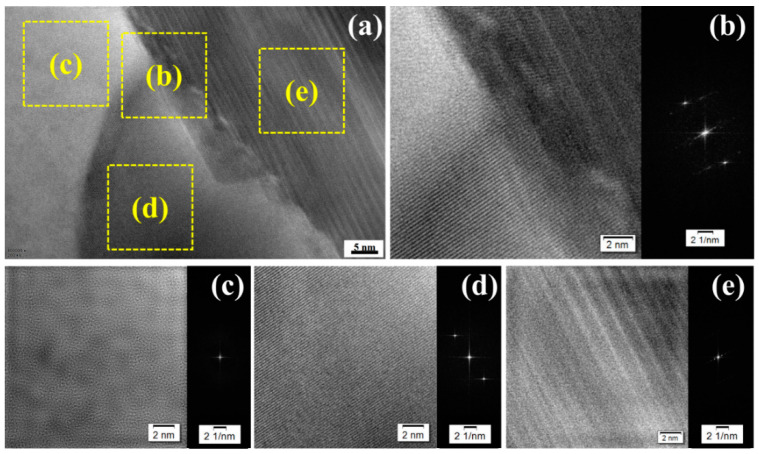
High-resolution transmission electron microscopy (HRTEM) images of the SiC/Al boundary in the TLSPed-30 J specimen. (**a**) Full-scale view of the SiC/Al boundary, (**b**) inverse fast Fourier transform (IFFT) image taken from the square (b) showing a metallurgical combining interface, (**c**) IFFT image taken from the square (c) showing the enlarged view of amorphous structure, (**d**) IFFT image taken from the square (d) showing the enlarged view of uniform atom layer in the aluminum substrate and (**e**) IFFT image taken from the square (e) showing stacking faults inside the SiC particle.

Figure 6 expounds the high-resolution transmission electron microscopy (HRTEM) image of the SiC/Al boundary in the TLSPed-30 J specimen. As can be observed in Figure 6a, four typical zones can be divided, as depicted by the yellow squares (b), (c), (d) and (e). Figure 6b shows the inverse fast Fourier transform (IFFT) image taken from the square (b), which is exactly positioned at the SiC/Al boundary, indicating a metallurgical combining interface. This interface means that no microcrack could be easily generated. Figure 6c shows the IFFT image taken from the square (c), where an amorphous structure was obtained. Differently, Figure 6d shows the IFFT image taken from the square (d), where a uniform atom layer can be observed, which corresponds to the aluminum substrate. Figure 6e shows the IFFT image taken from the square (e), which was placed at the SiC particle. Note that stack fault was generated. These four distinct zones reveal that different deformation mechanisms simultaneously occurred during the peening process.

### 3.2. Microhardness

**Figure 7:**
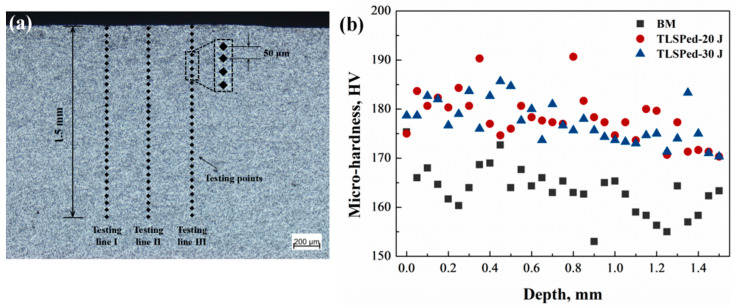
(**a**) The demonstration of microhardness measurement in the BM and LSPed specimens. (**b**) Microhardness distributions in the depth direction measured on the cross-section of BM, TLSPed-20 J and TLSPed-30 J specimens.

Figure 7a demonstrates the detailed measurement of the specimens, in which the microhardness was measured down to 1.5 mm with a step distance of 50 μm. The measurements were conducted on three parallel testing lines, and the averaged value was presented. Figure 7b presents the microhardness distribution in the depth direction measured on the cross-section of BM, TLSPed-20 J and TLSPed-30 J specimens. The error bar was not included, so as to clearly describe the value of each measured point. The original microhardness obtained from the BM specimen fluctuated between 155 and 170 HV. The randomly distributed SiC particles in the aluminum substrate can be taken as one possible reason for this fluctuation. The microhardness in both TLSPed-20 J and TLSPed-30 J specimens fell in the range of 170 to 185 HV. The increase in the microhardness can be mainly attributed to the generation of high-density dislocations. According to the Hall-Petch theory [41], microhardness value, HV, can be expressed by the following:

(3) $$HV=HV_{0}+aGb\rho^{1/2}$$

where *HV*_0_ is the microhardness of an ideal material without any defects, *a* is a constant of the material, *G* is the shearing modulus, *b* is the Burgers vector and *ρ* is the dislocation density. With high densities of dislocations generated in the LSPed specimens, the enhancement in the surface layer was achieved as expected. Moreover, the microhardness values in the TLSPed-20 J and TLSPed-30 J specimens did not show a distinct difference, which can be attributed to the saturation of the LSP-introduced dislocation density. The randomly distributed SiC particle could also be a reason for the microhardness increase, as the indenter probe will be inevitably loaded on the SiC particles. It is also worth noting that the attenuation of the microhardness is not obvious, which indicates that the laser shock peening affected layer in the SiC/Al composite was thicker than 1.5 mm. Peyre et al. also reported a similar result that LSP treatment is a feasible method to obtain a higher microhardness and larger depth of the hardened layer [42].

### 3.3. Residual Stress

**Figure 8:**
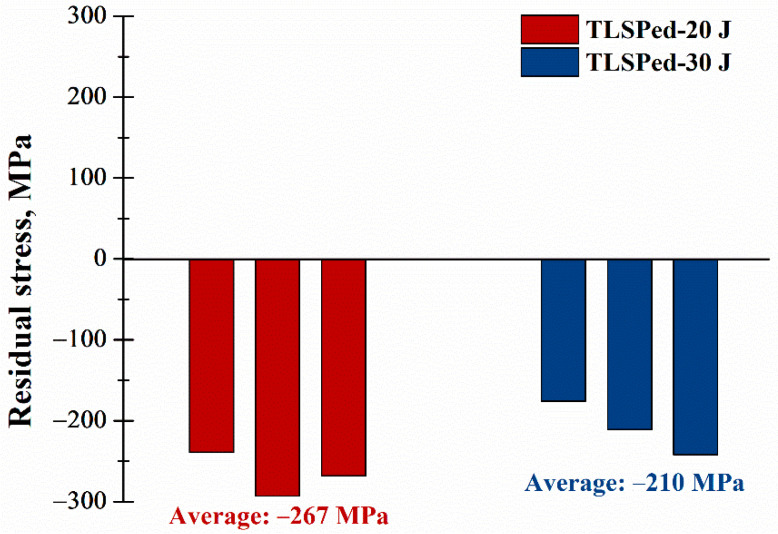
Compressive residual stress measured on the surface of the TLSPed-20 J and TLSPed-30 J specimens.

Figure 8 exhibits the surface compressive residual stress measured on the TLSPed-20 J and TLSPed-30 J specimens with averaged residual stress values of 267 and 210 MPa, respectively. Moreover, uniform stress distributions were achieved in both cases, which can be attributed to the relatively higher overlapping rate (40%) and the multiple peening times (peened twice). It is additionally notable that the compressive residual stress value in the TLSPed-30 J specimen was smaller than that in the TLSPed-20 J specimen. This means that using higher energy to peen a metal with relatively low yield strength may introduce a lower value of residual stress. This phenomenon can be linked to the stress hole effect and stress saturation effect [43,44]. Both effects can generate permanent reverse deformation and cause a decrease in residual stress on the top surface.

**Figure 9:**
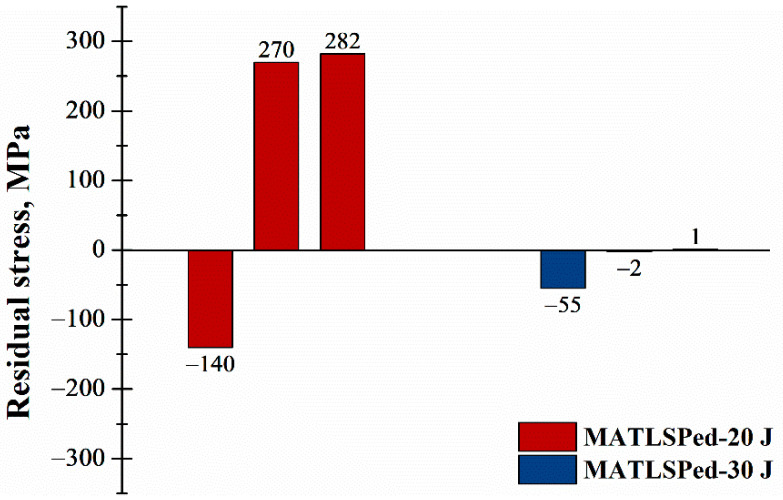
Residual stress measured on MATLSPed-20 J and MATLSPed-30 J specimens after milling down 50 μm from the original peened surface.

Figure 9 describes the residual stress after milling measured on MATLSPed-20 J and MATLSPed-30 J specimens. The measurements were carried out on the surfaces, where a 50 μm thick layer was milled. Compared with their original stress values, the compressive residual stresses of both specimens were significantly decreased, even resulting in a tensile state. Moreover, the change of residual stress in the 30 J peened specimen was less severe than that of the 20 J peened specimen. The reason for this can be described as follows. First, the stress hole effect induced a maximum stress value on the subsurface, but not the top surface of the specimen, which led to the residual stress of 30 J peened specimen in the 50 μm layer having a larger stress value than that of the 20 J peened specimen. Second, when the milling was conducted, the existed SiC particles increased the friction, which led to a larger thermal effect and oxidation. The milled surface appeared to be a dark gray color, and hence tensile stress was introduced. Similar surface tensile residual stress states have also been reported [45,46]. The tensile stress neutralized the original compressive residual stress. Finally, when it had a higher compressive residual stress, the neutralized stress could still stay compressive (see MATLSPed-30 J specimen); otherwise, it could also turn into tensile stress, as shown in the MATLSPed-20 J specimen.

### 3.4. Three-Point Bending Fatigue

**Figure 10:**
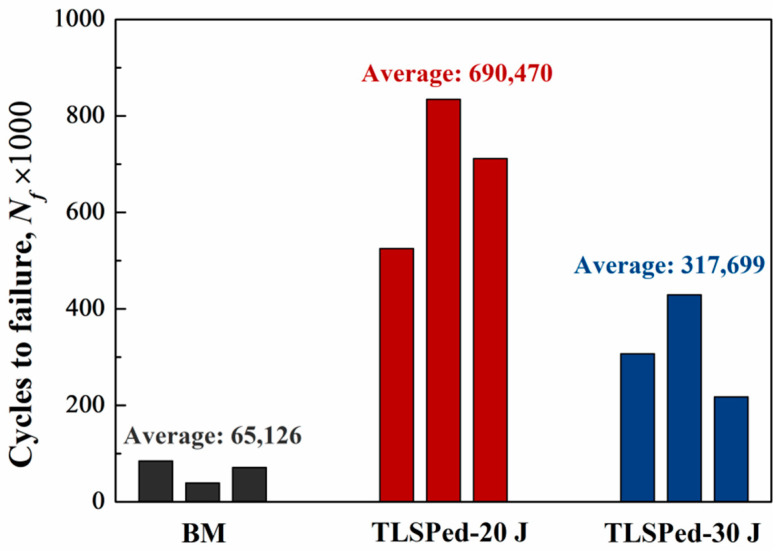
Three-point bending fatigue properties of BM, TLSPed-20 J and TLSPed-30 J specimens.

Figure 10 displays the three-point bending fatigue properties of BM, TLSPed-20 J and TLSPed-30 J specimens. It can be seen that LSP played a significant role in enhancing the fatigue life of the SiCp/2009Al composite. The fatigue life of the BM specimen was 65,126 cycles, while the fatigue lives of TLSPed-20 J and TLSPed-30 J specimens were 690,470 and 317,699 cycles, being 10.60 and 4.88 times higher than that of the BM specimens, respectively. Additionally, as indicated by the residual stress results in Figure 8, surface compressive residual stress in the TLSPed-20 J specimen is higher than that in the TLSPed-30 J specimen, which can provide a higher resistance for the crack growth, and minimize the local stress level during cycling loading [47]. Such results also indicate that the effectiveness of LSP is not linear to the laser pulse energy, and laser pulse energy that is too high could even generate a negative effect on the fatigue life. Hence, the selection of laser parameters for peening metal matrix composite is a key factor in achieving the desired fatigue property.

**Figure 11:**
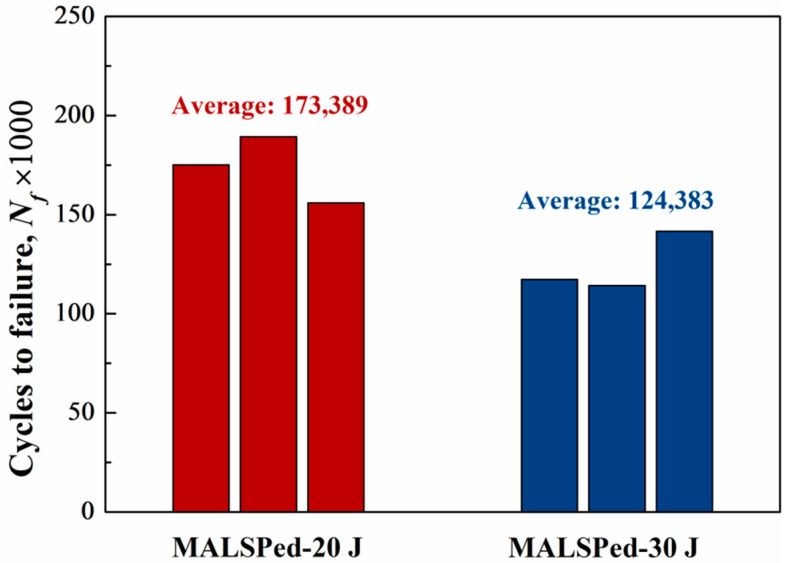
Three-point bending fatigue properties of MATLSPed-20 J and MATLSPed-30 J specimens.

Figure 11 demonstrates the three-point bending fatigue properties of specimens after milling down 50 μm from the original peened surface (TLSPed-20 J and TLSPed-30 J). It can be seen that the fatigue lives of MATLSPed-20 J and MATLSPed-30 J specimens were 173,389 and 124,383 cycles, respectively. Compared with the original 20 J and 30 J peened specimens, the fatigue life decreased by 75% and 61%. However, they are still 2.66 and 1.91 times higher than that of the BM specimens. These significant decreases can be mainly associated with residual stress change, as a decrease in the compressive residual stress can weaken the effectiveness of the peening. Additionally, the change in geometry could be another reason, as a decrease in the thickness of the specimen can lead to an increase in the overall stress level. However, the effect of geometry change should be minor, as the machining can also introduce a slight difference in the specific size. Hence, residual stress is particularly important in obtaining a better fatigue property for metal matrix composite.

## 4. Conclusions

LSP was applied to a SiC particle reinforced 2009 aluminum composite. The microstructure, microhardness and residual stress evolutions were investigated accordingly, and the fatigue properties of three-point bending were further evaluated. The main findings are summarized as follows:

1. TEM observation shows that LSP introduced a large number of dislocations in the aluminum substrate, but caused stacking faults in the SiC particles of the SiCp/2009Al composite. HRTEM investigation indicates that no possible crack or any other defect was generated at the SiC/Al interface.
2. LSP provided a hardening for the near-surface layer of the SiCp/2009Al composite. The microhardness value increased from 155–170 HV to 170–185 HV, with an affected depth of more than 1.5 mm, after 20 and 30 J twice peening.
3. Compressive residual stresses of a value more than 200 MPa were introduced into the surface of SiCp/2009Al composite after 20 and 30 J twice peening, while the compressive residual stresses decreased after surface milling, even resulting in a tensile state in 20 J twice peened specimens.
4. Fatigue lives were significantly increased after 20 and 30 J twice peening, which are 10.60 and 4.88 times higher than that of the BM specimens. The milling of the peened surface resulted in a partial decrease in the fatigue lives, while they were still 2.66 and 1.91 times higher than those of the BM specimens.

This study is expected to provide new insight into the understanding of the surface modification of ceramic reinforced metal composite for both science and engineering aspects. The results in this study can benefit both the field of laser shock peening and ceramic reinforced metal composite manufacturing.

## Figures and Tables

**Table 1:** Chemical compositions of 2009 aluminum alloy employed in this study (wt%).

Element	Cu	Mg	Si	Fe	Zn	O	Al
Percentage	3.2–4.4	1.0–1.9	0.25	0.2	0.1	0.6	Bal.

**Table 2:** Chemical compositions and possible phases of the Cu enrichment in Figure 3a (at. %).

Element	Al	Cu	Possible phase
Point 1	61.44	38.56	Al_3_Cu_2_
Point 2	44.75	55.25	AlCu

## Data Availability

The data presented in this study are available upon request from the corresponding author.
